# Supplementary material for: Host Range Evolution of Potyviruses: A Global Phylogenetic Analysis
Source: Viruses. 2020 Jan 16;12(1):111. doi: 10.3390/v12010111 (PMC7020010; doi:10.3390/v12010111)
Supplement: Supplementary file 1 [file viruses-12-00111-s001.zip › Figure S1.pdf]

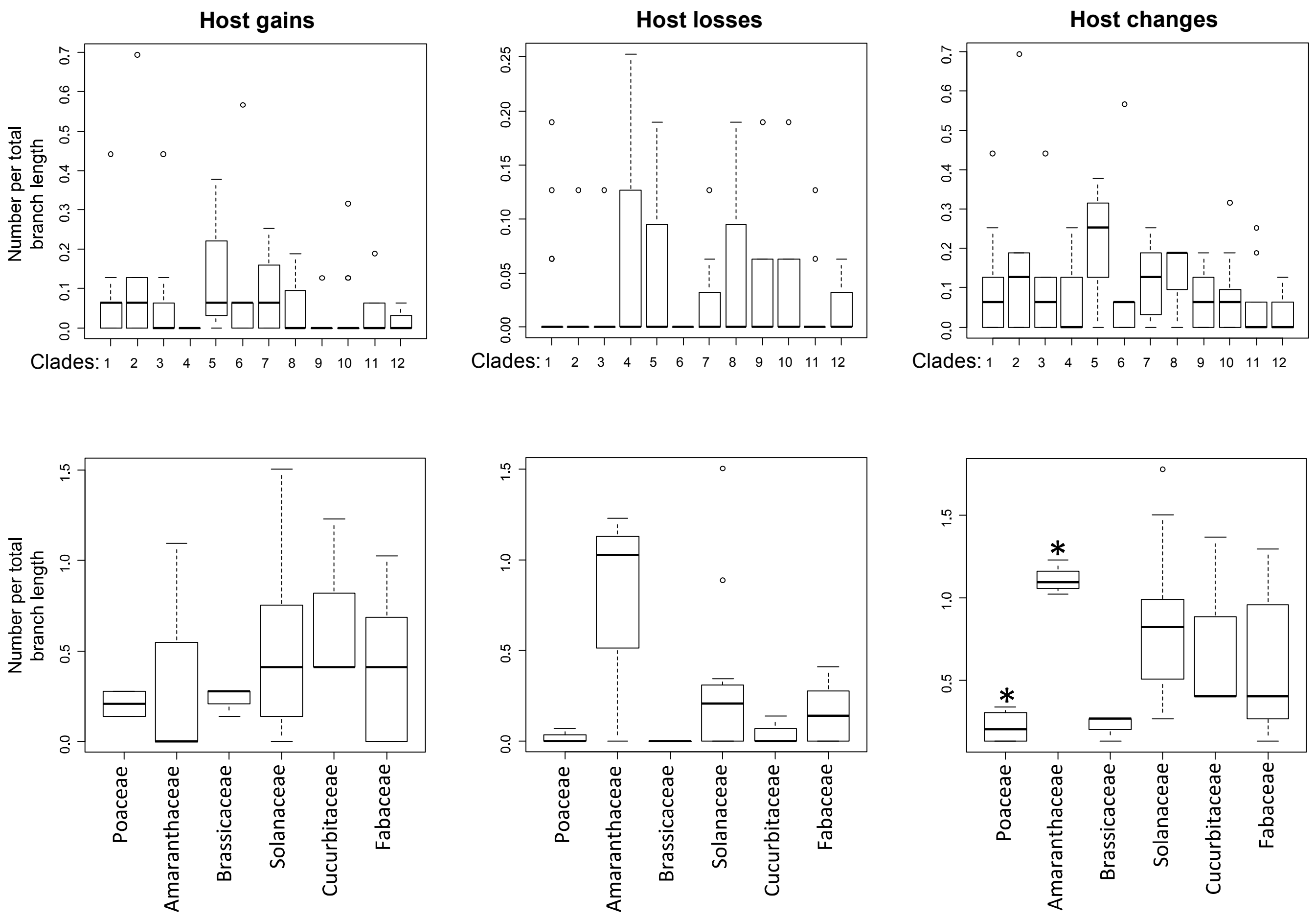

Figure S1. Distribution of host gains, host losses or total host changes across potyvirus clades (Fig. 1) and across plant families. For potyvirus clades, the different branches were considered as replicates. For plant families, the different species were considered as replicates. Multiple Kruskal-Wallis tests were used to compare the numbers of host/non-host status changes per total branch length across the compared groups. The asterisks indicate the single pairwise comparison that revealed a significant difference (p-value<0.05).
