# Supplementary material for: Host Range Evolution of Potyviruses: A Global Phylogenetic Analysis
Source: Viruses. 2020 Jan 16;12(1):111. doi: 10.3390/v12010111 (PMC7020010; doi:10.3390/v12010111)
Supplement: Supplementary file 1 [file viruses-12-00111-s001.zip › Table S2.docx]

**Table S2.** Inferred host changes corresponding to the different branches in the potyvirus phylogenetic tree (Fig. 1). Branches with no changes and changes for which alternative scenarios exist are omitted.

| **Branch name or number** | **Host plant gains** | **Host plant losses** |
| --- | --- | --- |
| AmLMV | *Phaseolus vulgaris*, *Vicia faba* | *Nicotiana glutinosa* |
| AV-1 | *Beta vulgaris* | *Nicotiana benthamiana*, *Nicotiana clevelandii*, *Nicotiana sylvestris* |
| BCMV |  | *Lathyrus odoratus* |
| BiMoV | *Lactuca sativa* | *Capsicum frutescens*, *Nicotiana glutinosa*, *Trigonella foenum-graecum* |
| BiMV | *Lactuca sativa* |  |
| BtMV | *Beta vulgaris*, *Nicotiana glutinosa* |  |
| BYMV | *Cucumis melo, Medicago sativa* |  |
| CeMV | *Apium graveolens* |  |
| ChiVMV |  | *Chenopodium amaranticolor*, *Chenopodium quinoa* |
| CSV | *Triticum aestivum* |  |
| DsMV |  | *Nicotiana clevelandii, Phaseolus vulgaris*, *Vicia faba* |
| EuRSV |  | *Nicotiana clevelandii* |
| FreMV |  | *Nicotiana benthamiana*, *Phaseolus vulgaris*, *Vicia faba* |
| HiMV | *Cucumis sativus*, *Datura stramonium*, *Nicotiana glutinosa*, *Solanum lycopersicum* | *Nicotiana benthamiana* |
| HMV | *Datura stramonium*, *Nicotiana glutinosa* | *Trigonella foenum-graecum* |
| HyaMV |  | *Chenopodium amaranticolor*, *Chenopodium quinoa*, *Nicotiana clevelandii* |
| ISMV |  | *Chenopodium quinoa* |
| KoMV |  | *Chenopodium amaranticolor*, *Chenopodium quinoa*, *Nicotiana benthamiana*, *Nicotiana clevelandii* |
| LMV | *Beta vulgaris*, *Lactuca sativa*, *Phaseolus vulgaris* |  |
| LYSV | *Allium cepa* | *Nicotiana clevelandii*, *Nicotiana megalosiphon* |
| NYSV | *Glycine max* |  |
| OrMV |  | *Chenopodium amaranticolor* |
| OYDV | *Allium cepa* | *Nicotiana clevelandii* |
| PeMoV | *Cucumis sativus*, *Vigna unguiculata* |  |
| PepSMV | *Solanum lycopersicum* |  |
| PkMV |  | *Nicotiana clevelandii* |
| PPV | *Cucumis sativus*, *Nicotiana glutinosa*, *Solanum lycopersicum*, *Trifolium pratense*, *Trifolium repens*, *Vigna unguiculata* |  |
| PRSV |  | *Lathyrus odoratus, Lupinus albus*, *Nicotiana clevelandii* |
| PSbMV | *Beta vulgaris, Brassica campestris*, *Brassica oleracea*, *Catharanthus roseus*, *Medicago sativa*, *Vicia faba* |  |
| PVA | *Solanum tuberosum* |  |
| PVMV | *Catharanthus roseus*, *Datura stramonium*, *Nicotiana glutinosa*, *Solanum melongena* |  |
| PVV | *Solanum tuberosum* |  |
| PVY | *Apium graveolens*, *Beta vulgaris*, *Cucumis sativus*, *Solanum lycopersicum*, *Solanum melongena, Solanum tuberosum, Vigna unguiculata* |  |
| SCMV | *Glycine max*, *Hordeum vulgare*, *Phaseolus vulgaris* | *Lathyrus odoratus* |
| SPFMV |  | *Chenopodium quinoa* |
| SPLV |  | *Nicotiana sylvestris* |
| TEV | *Beta vulgaris*, *Capsicum annuum*, *Capsicum frutescens*, *Nicotiana glutinosa*, *Solanum lycopersicum*, *Solanum melongena*, *Solanum tuberosum* |  |
| TuMV | *Beta vulgaris, Brassica campestris*, *Brassica oleracea*, *Cucumis sativus*, *Datura stramonium*, *Lactuca sativa*, *Nicotiana glutinosa*, *Raphanus sativus*, *Vicia faba* |  |
| TVMV |  | *Chenopodium quinoa*, *Nicotiana clevelandii* |
| WMV | *Catharanthus roseus*, *Cucumis sativus*, *Datura stramonium*, *Nicotiana glutinosa*, *Trifolium pratense* |  |
| WVMV |  | *Vigna unguiculata* |
| ZYMV |  | *Glycine max* |
| 72 | *Capsicum frutescens*, *Nicotiana glutinosa* |  |
| 75 | *Catharanthus roseus* |  |
| 77 | *Solanum lycopersicum* |  |
| 79 |  | *Chenopodium amaranticolor*, *Chenopodium quinoa* |
| 82 | *Beta vulgaris*, *Citrullus lanatus*, *Cucumis sativus*, *Glycine max*, *Medicago sativa*, *Nicotiana glutinosa*, *Phaseolus vulgaris*, *Trifolium pratense*, *Trifolium repens*, *Vicia faba*, *Vigna unguiculata* |  |
| 84 | *Datura stramonium* |  |
| 86 | *Nicotiana glutinosa*, *Solanum lycopersicum* |  |
| 98 | *Phaseolus vulgaris* |  |
| 100 | *Capsicum annuum, Solanum lycopersicum*, *Solanum tuberosum* |  |
| 102 | *Capsicum frutescens* |  |
| 104 | *Citrullus lanatus*, *Cucumis melo*, *Cucumis sativus* |  |
| 105 | *Citrullus lanatus*, *Glycine max*, *Phaseolus vulgaris*, *Vicia faba* |  |
| 107 |  | *Chenopodium quinoa* |
| 108 | *Cucumis melo*, *Medicago sativa* |  |
| 109 | *Cucumis sativus*, *Vigna unguiculata* |  |
| 113 |  | *Cucumis sativus* |
| 117 | *Zea mays* | *Chenopodium amaranticolor*, *Chenopodium quinoa* |
| 123 | *Hordeum vulgare* |  |
