# Supplementary material for: Host Range Evolution of Potyviruses: A Global Phylogenetic Analysis
Source: Viruses. 2020 Jan 16;12(1):111. doi: 10.3390/v12010111 (PMC7020010; doi:10.3390/v12010111)
Supplement: Supplementary file 1 [file viruses-12-00111-s001.zip › Table S3.docx]

**Table S3.** Branches in the potyvirus phylogenetic tree where the number of inferred host gains significantly exceeded expectations of random distributions.

| **Branch**  **(Fig. 1)** | **Number of inferred host gains (Table 3)** | **Expected numbers of host gains among 10000 Monte Carlo simulations** | | |
| --- | --- | --- | --- | --- |
|  |  | **95% confidence interval (CI)** | **99% CI** | **99.9% CI** |
| PPV | 6 | 0-4* | 0-5* | 0-7 |
| PVMV | 4 | 0-3* | 0-3* | 0-5 |
| PVY | 7 | 0-3* | 0-4* | 0-5* |
| SCMV | 3 | 0-2* | 0-3 | 0-4 |
| TEV | 7 | 0-5* | 0-6* | 0-8 |
| TuMV | 9 | 0-4* | 0-4* | 0-6* |
| WMV | 5 | 0-2* | 0-2* | 0-3* |
| 82 | 11 | 0-5* | 0-6* | 0-7* |
| 105 | 4 | 0-3* | 0-4 | 0-5 |

* Indicates cases where the number of inferred host gains was higher than the upper bound of the CI of Monte Carlo simulations.
