# Supplementary material for: Host Range Evolution of Potyviruses: A Global Phylogenetic Analysis
Source: Viruses. 2020 Jan 16;12(1):111. doi: 10.3390/v12010111 (PMC7020010; doi:10.3390/v12010111)
Supplement: Supplementary file 1 [file viruses-12-00111-s001.zip › Table S4.docx]

**Table S4.** Co-occurrence of host losses (*i.e.* host losses inferred on the same tree branch) or host gain and loss that were found multiple times (*i.e.* on at least two different tree branches for pairs of plant species).

| **Plant species 1** | **Plant species 2** | **Branch name or number**  **(Fig. 1)** | **Number of independent host status change co-occurrences** |
| --- | --- | --- | --- |
| **Co-occurrence of host losses** | |  |  |
| *Nicotiana benthamiana* | *Nicotiana clevelandii* | AV-1, KoMV | 2 |
| *Chenopodium amaranticolor* | *Chenopodium quinoa* | ChiVMV, HyaMV, KoMV, 79, 117 | 5 |
| *Phaseolus vulgaris* | *Vicia faba* | DsMV, FreMV | 2 |
| **Co-occurrence of host gain and loss** | |  |  |
| *Allium cepa* | *Nicotiana clevelandii* | LYSV, OYDV | 2 |
